# Pneumatosis intestinalis associated with lenvatinib during thyroid cancer treatment: a case report

**DOI:** 10.1186/s13256-021-03158-w

**Published:** 2021-11-12

**Authors:** Soji Toda, Hiroyuki Iwasaki, Daisuke Murayama, Maya Isoda, Hirotaka Nakayama, Nobuyasu Suganuma, Katsuhiko Masudo

**Affiliations:** 1 Department of Breast and Endocrine Surgery, Kanagawa Cancer Center, 2-3-2 Nakao, Asahi-ku, Yokohama, 241-8515 Japan; 2 Department of Surgery, Hiratsuka Kyosai Hospital, Hiratsuka, Japan; 3 Department of Surgery, Yokohama City University School of Medicine, Yokohama, Japan; 4 Department of Breast and Thyroid Surgery, Yokohama City University Medical Center, Yokohama, Japan

**Keywords:** Lenvatinib, Vascular endothelial growth factor, Pneumatosis intestinalis, Thyroid cancer, Case report

## Abstract

**Background:**

Pneumatosis intestinalis is a rare disease characterized by gas-filled cysts within the submucosa or serosa of the intestinal tract. In recent years, pneumatosis intestinalis was reported in patients undergoing cancer treatment, and some case reports exist that report that pneumatosis intestinalis occurs during administration of vascular endothelial growth factor inhibitors, such as bevacizumab and sunitinib. Here, we report the first case of pneumatosis intestinalis during lenvatinib treatment.

**Case presentation:**

A 77-year-old Japanese man presented to our hospital with a chief complaint of numbness in the right leg and weakness of the lower limbs 9 years after right thyroid lobectomy. Computed tomography showed a tumor 90 mm in size from the lumbar spine to the sacrum, causing spinal cord compression. Blood tests showed that the patient’s thyroglobulin level was increased to 11,600 ng/ml. We diagnosed him with thyroid cancer with bone metastases. External beam radiotherapy (39 Gy/13 Fr) was performed on the bone metastases, followed by total thyroidectomy and radioactive iodine therapy. Four months after radioactive iodine therapy, lenvatinib was introduced because the symptoms of numbness and weakness recurred. Lenvatinib was introduced at dose of 24 mg, and then it was reduced to 14 mg owing to Common Terminology Criteria for Adverse Event grade 3 paronychia of the right foot. Although no further significant adverse events occurred, a scheduled computed tomography image showed pneumatosis intestinalis of the ascending colon 14 weeks after the introduction of lenvatinib. No abdominal or digestive symptoms were observed; therefore, we selected conservative treatment. We discontinued lenvatinib for a week, but we were required to restart lenvatinib as the numbness in the right leg worsened after withdrawal. Since the introduction of lenvatinib, 3 years and 5 months passed; we continued lenvatinib treatment, and the therapeutic effect remains partial response. There has been no recurrence of pneumatosis intestinalis.

**Conclusions:**

Although rare, it is important to recognize that pneumatosis intestinalis can occur in association with lenvatinib and should be differentiated from intestinal perforation. Pneumatosis intestinalis association with lenvatinib can be improved by withdrawal.

## Background

Pneumatosis intestinalis (PI) is a rare disease characterized by gas-filled cysts within the submucosa or serosa of the intestinal tract [1]. Many patients are asymptomatic, but PI can cause symptoms such as vomiting, bloating, diarrhea, and abdominal pain. It can be incidentally identified by computed tomography (CT) imaging [2].

In recent years, PI was reported in patients undergoing cancer treatment, and some case reports exist that report that PI occurs during administration of vascular endothelial growth factor (VEGF) inhibitors, such as bevacizumab and sunitinib. Here, we report a case of PI that developed during lenvatinib treatment for thyroid cancer, which is a vascular growth factor receptor tyrosine kinase inhibitor.

## Case presentation

A 77-year-old Japanese man presented with a follicular thyroid tumor, and the blood thyroglobulin level was 1800 ng/ml. He had no medical history or medications, but his mother and brother had history of colorectal carcinoma and prostate carcinoma. He had smoked three cigarettes for 3 years, and he did not consume alcohol regularly. He received right thyroid lobectomy, and the pathological examination showed no malignant findings such as vascular invasion or capsular invasion. The thyroglobulin level decreased to 14 ng/ml postoperatively. Follow-up at our hospital was discontinued.

**Fig. 1:**
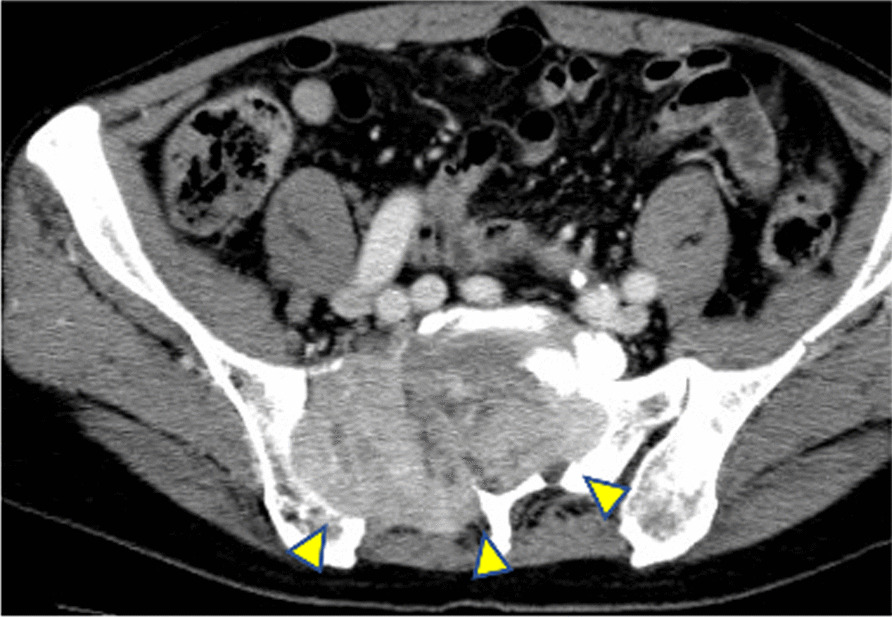
CT image showing 90-mm bone metastasis (indicated by arrows) causing spinal cord compression

Nine years after the operation, he presented to our hospital again with numbness of the right leg and difficulty of walk. On examination, weakness of the right lower limbs was observed but no mass was palpable on the lower back and lower limbs. CT showed a tumor 90 mm in size from the lumbar spine to the sacrum, causing spinal cord compression (Fig. 1). Blood tests showed that the thyroglobulin level was increased to 11,600 ng/ml. Ultrasonography of thyroid showed a 14 mm iso-echoic mass in the residual left lobe suggesting follicular tumor. We diagnosed him with thyroid cancer with bone metastases. External beam radiotherapy (39 Gy/13 Fr) was performed on the bone metastases, followed by total thyroidectomy and radioactive iodine therapy (RAI; 131-I 100 mCi).

**Fig. 2:**
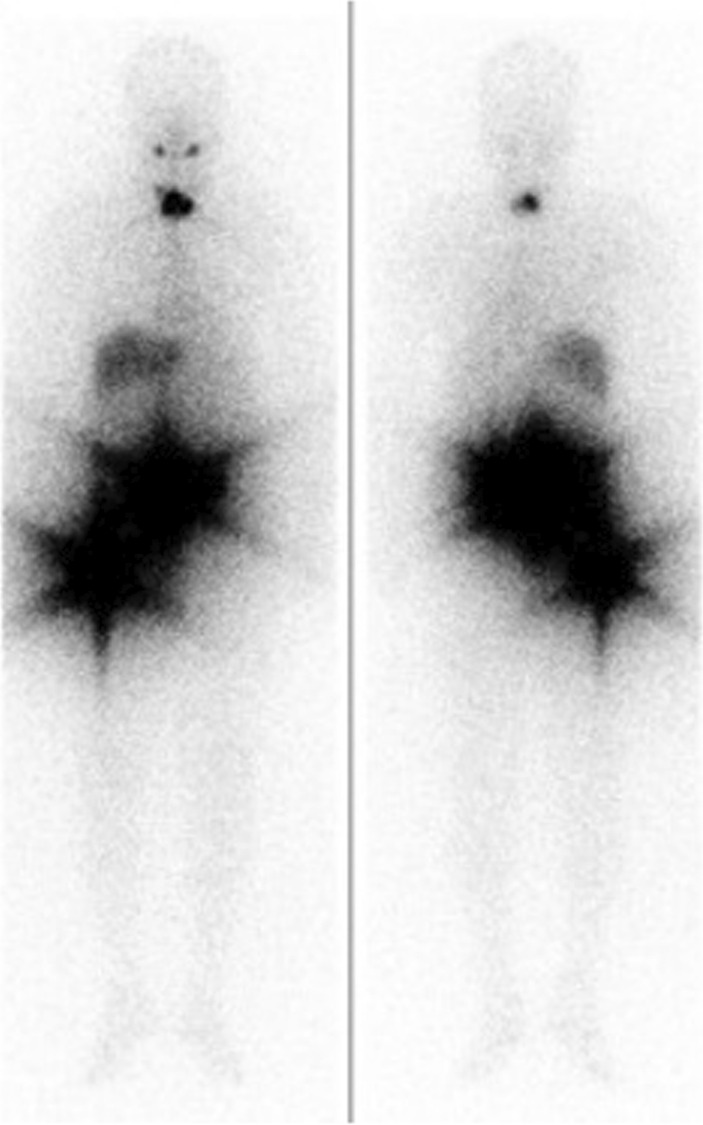
Scintigraphy of RAI therapy showing high accumulation on the pelvis

The pathology of the residual thyroid gland was follicular tumor. Although we examined the whole thyroid, including the previous specimen of right lobe, we could not find any malignant features such as vascular invasion or capsular invasion. However, we diagnosed follicular thyroid carcinoma owing to the presence of bone metastasis. Scintigraphy of RAI therapy showed high accumulation on the right pelvis and the thyroid bed (Fig. 2). The numbness in the right thigh and weakness of the lower limbs improved after the start of treatment, and the thyroglobulin level decreased to 3940 ng/ml. However, 4 months after RAI therapy, the symptoms of numbness and weakness in the lower extremities recurred. The tumor size of pelvic bone metastasis was re-increased, and the thyroglobulin level increased to 5270 ng/ml. The patient was diagnosed with RAI-resistant thyroid follicular cancer, and lenvatinib was introduced.

**Fig. 3:**
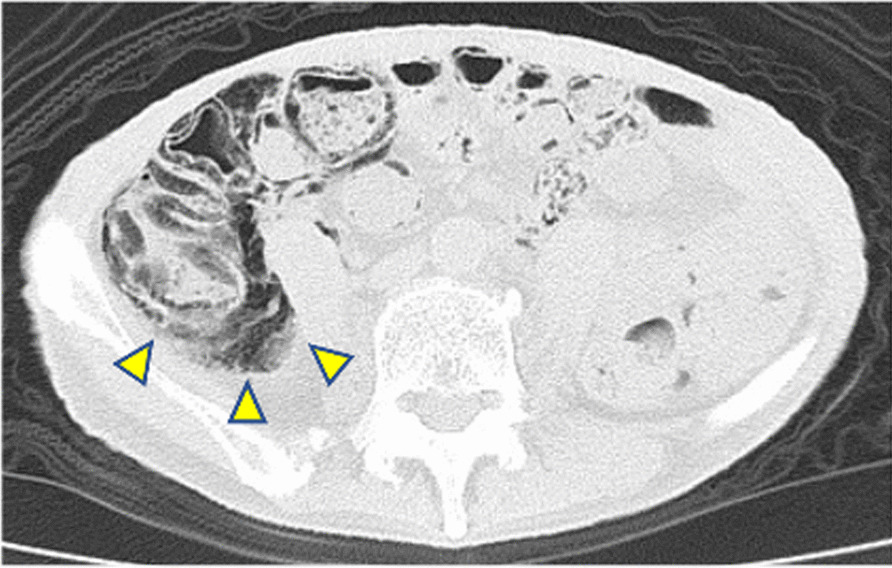
CT image taken 14 weeks after the introduction of lenvatinib showing pneumatosis intestinalis of the ascending colon. The arrows are pointing to emphysema in the intestinal wall of the ascending colon

Lenvatinib was introduced at dose of 24 mg per oral once daily, and he was started on calcium blocker and angiotensin II receptor blocker because of hypertension. Two weeks later, he developed National Cancer Institute (NCI) Common Terminology Criteria for Adverse Event (CTCAE) grade 3 paronychia of the right foot, which was improved by partial nail avulsion, intravenous administration of ceftriaxone, and discontinuation of lenvatinib for 3 weeks. Lenvatinib was reduced to 14 mg and resumed. Although no significant adverse events occurred after dose reduction, emphysema was found in the intestinal wall of the ascending colon on a scheduled CT image taken 14 weeks after the introduction of lenvatinib to determine the therapeutic effect (Fig. 3).

The patient visited our hospital 9 days after the CT examination as planned. He had no abdominal or digestive symptoms. On examination, temperature was 36.8 °C, blood pressure 115/75 mmHg, and pulse 63 beats per minute. Physical examination of neck and abdomen was normal, and neurological abnormalities of the legs were not observed. Laboratory testing revealed a white cell count of 4400/μL (reference range 3900–9800/μL), hemoglobin level of 11.4 g/dL (reference range 13.5–17.6 g/dL), and platelet count of 279,000/μL (reference range 131,000–362,000/μL). Blood levels of electrolytes and C-reactive protein were normal, as were results of tests for renal function and liver function. Urinalysis was normal, including urine protein. Re-examination of CT was performed, and it showed the air in the intestinal wall was reduced. Because there were no findings suggestive of intestinal ischemia or perforation, he was diagnosed with pneumatosis intestinalis and lenvatinib was discontinued. He was not hospitalized and did not need any medications.

**Fig. 4:**
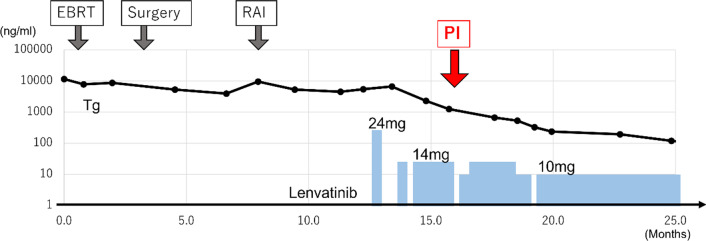
Clinical course. The graph shows the transition of thyroglobulin, the timing of lenvatinib treatment, and the dose. The times of external beam radiotherapy (EBRT), surgery, and radioactive iodine (RAI) are shown above the graph. The red arrow indicates the time of pneumatosis intestinalis (PI). The thyroglobulin level was decreased after introduction of lenvatinib, reflecting the anti-tumor effect of lenvatinib. PI occurred 14 weeks after the introduction, and it was improved by 1-week withdrawal of lenvatinib

The numbness of the right leg worsened after withdrawal of lenvatinib, so the patient was required to restart lenvatinib at a dose of 10 mg after a week of withdrawal. Three weeks later, we tried increasing the dose of lenvatinib to 14 mg. However, we needed to reduce back to 10 mg because of anorexia (Fig. 4). Two months after the diagnosis of pneumatosis intestinalis, CT showed that the emphysema of the intestinal tract had completely disappeared. Three years and 5 months passed since the introduction of lenvatinib; we continued lenvatinib treatment, and the therapeutic effect remains partial response. There was no recurrence of PI.

## Discussion

This is the first case report of PI during lenvatinib treatment. In the present case, the patient had no symptoms and physical examinations were normal. PI was detected on a scheduled CT scan during lenvatinib treatment and improved by withdrawal of lenvatinib. Recently, there have been some case reports of PI caused by VEGF inhibitors such as bevacizumab, sunitinib, and sorafenib. This case showed that lenvatinib, a VEGF inhibitor, can also cause PI, and PI association with lenvatinib can be improved by withdrawal.

Lenvatinib is an orally available multitargeted tyrosine kinase inhibitor that targets VEGF receptors 1–3, fibroblast growth factor receptors 1–4, platelet-derived growth factor receptor-α, and the RET proto-oncogene and KIT proto-oncogene receptor tyrosine kinase, and it exhibits an antitumor effect by inhibiting angiogenesis [3, 4]. In a phase III study of radioiodine refractory differentiated thyroid carcinoma (SELECT study), lenvatinib improved the progression-free survival significantly over placebo [5].

Pneumatosis intestinalis is a rare disease characterized by subserosal or submucosal gas-filled cysts of the digestive tract. Categories of relevant factors are mechanical, inflammatory, autoimmune, infectious, pulmonary, and drug-induced. It is often found by CT imaging. Researchers reported that 70% of patients of PI are asymptomatic [2], although it may manifest as a more serious condition such as intestinal ischemia; its mostly asymptomatic nature, however, makes determining the overall incidence of PI difficult. Although perforation of the colon can be a fatal course, many PI cases improve conservatively. However, surgical treatment should be considered in cases of obstructive symptoms, such as vomiting, nausea, and pain, or findings such as white blood cells > 12,000/mm^3^ and portal venous gas on CT [1].

In this case, no other risk factors for PI were present, such as diabetes, steroid drugs, underlying colon disease, or preexisting acute colon infection. PI was incidentally detected on a scheduled CT, and the patient presented with no abdominal symptoms. PI was managed conservatively and resolved.

No reports of PI related to lenvatinib are found in the literature. However, researchers report that PI is related to cancer therapeutic agents. Recently, there have been some reports of PI cases related to molecular target drugs such as VEGF inhibitors. Bevacizumab, a monoclonal antibody to VEGF-A, was found to significantly reduce the capillary density of the small intestinal villi. Poor density of blood vessels may result in diminished intestinal mucosal regeneration and microperforation in the intestinal wall [6, 7]. Not only bevacizumab but also multitargeted tyrosine kinase inhibitors, such as sunitinib and sorafenib, have been reported to be related to PI [8–10]. The detailed mechanism of PI related to multitargeted tyrosine kinase inhibitors is unknown.

It is necessary to note that rebounds have been reported during withdrawal of anti-VEGF drugs [11, 12]. In our patient, when lenvatinib was discontinued for a week, neurological symptoms due to the thyroid cancer were aggravated. In Japan, lenvatinib and sorafenib are approved for differentiated thyroid cancer, and no other option than VEGF inhibitors exists. Therefore, we decided to readminister lenvatinib after PI. Since then, PI has not reoccurred, and the therapeutic effect of lenvatinib remained partial response for 3 years and 5 months.

## Conclusion

This is the first case report of PI during lenvatinib treatment. We experienced a rare case of PI related to lenvatinib that was detected on a scheduled CT. PI improved after discontinuation of lenvatinib. Knowing that PI can occur in association with lenvatinib and that it should be differentiated from intestinal perforation is important. PI association with lenvatinib can be improved by withdrawal.

## Data Availability

The data that support the findings of this study are available from the corresponding author upon reasonable request.

## Abbreviations

PI: Pneumatosis intestinalis
CT: Computed tomography
VEGF: Vascular endothelial growth factor
Fr: Frequency
RAI: Radioactive iodine
CTCAE: National Cancer Institute Common Terminology Criteria for Adverse Event

## References

[1] Greenstein AJ, *et al.* Pneumatosis intestinalis in adults: management, surgical indications, and risk factors for mortality. J Gastrointest Surg. 2007;11(10):1268–74. 10.1007/s11605-007-0241-9 17687617

[2] Shinagare AB, *et al.* Pneumatosis intestinalis and bowel perforation associated with molecular targeted therapy: an emerging problem and the role of radiologists in its management. Am J Roentgenol. 2012;199(6):1259–65. 10.2214/AJR.12.8782 23169717

[3] Matsui J, *et al.* E7080, a novel inhibitor that targets multiple kinases, has potent antitumor activities against stem cell factor producing human small cell lung cancer H146, based on angiogenesis inhibition. Int J Cancer. 2008;122(3):664–71. 10.1002/ijc.23131 17943726

[4] Matsui J, *et al.* Multi-kinase inhibitor E7080 suppresses lymph node and lung metastases of human mammary breast tumor MDA-MB-231 via inhibition of vascular endothelial growth factor-receptor (VEGF-R) 2 and VEGF-R3 kinase. Clin Cancer Res. 2008;14(17):5459–65. 10.1158/1078-0432.CCR-07-5270 18765537

[5] Schlumberger M, *et al.* Lenvatinib versus placebo in radioiodine-refractory thyroid cancer. N Engl J Med. 2015;372(7):621–30. 10.1056/NEJMoa1406470 25671254

[6] Kamba T, *et al.* VEGF-dependent plasticity of fenestrated capillaries in the normal adult microvasculature. Am J Physiol Heart Circ Physiol. 2006;290(2):H560–76. 10.1152/ajpheart.00133.2005 16172168

[7] Asmis TR, *et al.* Pneumatosis intestinalis: a variant of bevacizumab related perforation possibly associated with chemotherapy related GI toxicity. Invest New Drugs. 2008;26(1):95–6. 10.1007/s10637-007-9094-z 17962908

[8] Coriat R, *et al.* Pneumatosis intestinalis associated with treatment of cancer patients with the vascular growth factor receptor tyrosine kinase inhibitors sorafenib and sunitinib. Invest New Drugs. 2011;29(5):1090–3. 10.1007/s10637-010-9458-7 20524036

[9] Asahi Y, *et al.* Pneumatosis cystoides intestinalis secondary to sunitinib treatment for gastrointestinal stromal tumor. Case Rep Gastroenterol. 2018;12(2):432–8. 10.1159/000490657 PMC6120377 30186096

[10] Lee YS, *et al.* Pneumatosis cystoides intestinalis associated with sunitinib and a literature review. BMC Cancer. 2017;17(1):732. 10.1186/s12885-017-3744-0 PMC5679335 29121860

[11] Cacheux W, *et al.* Reversible tumor growth acceleration following bevacizumab interruption in metastatic colorectal cancer patients scheduled for surgery. Ann Oncol. 2008;19(9):1659–61. 10.1093/annonc/mdn540 18684697

[12] Mancuso MR, *et al.* Rapid vascular regrowth in tumors after reversal of VEGF inhibition. J Clin Invest. 2006;116(10):2610–21. 10.1172/JCI24612 PMC1578604 17016557

